# Higher Acid-Base Imbalance Associated with Respiratory Failure Could Decrease the Survival of Patients with Scrub Typhus during Intensive Care Unit Stay: A Gene Set Enrichment Analysis

**DOI:** 10.3390/jcm8101580

**Published:** 2019-10-02

**Authors:** Kyoung Min Moon, Kyueng-Whan Min, Mi-Hye Kim, Dong-Hoon Kim, Byoung Kwan Son, Youngha Oh, Woonyong Jung, Mijung Kwon, O-Yu Kwon

**Affiliations:** 1 Department of Internal Medicine, Gangneung Asan Hospital, University of Ulsan College of Medicine, Gangneung 25440, Korea; pulmogicu@gmail.com (K.M.M.); mymyee@empas.com (M.-H.K.); 2 Department of Pathology, Hanyang University Guri Hospital, Hanyang University College of Medicine, Guri 11923, Korea; yhoh@hanyang.ac.kr (Y.O.); pathjwy@hanyang.ac.kr (W.J.); 3 Departments of Pathology, Kangbuk Samsung Hospital, Sungkyunkwan University School of Medicine, Seoul 03181, Korea; idavid.kim@samsung.com; 4 Department of Internal Medicine, Eulji Hospital, Eulji University School of Medicine, Seoul 01830, Korea; sbk1026@eulji.ac.kr; 5 Department of Pathology, Hallym University Sacred Heart Hospital, Hallym University College of Medicine, Anyang 14068, Korea; mulank99@hallym.or.kr; 6 Departments of Medical Science and Anatomy and Cell Biology, College of Medicine, Chungnam National University, Daejeon 35015, Korea; oykwon@cnu.ac.kr

**Keywords:** scrub typhus, acid-base imbalance, intensive care unit, survival

## Abstract

Ninety percent of patients with scrub typhus (SC) with vasculitis-like syndrome recover after mild symptoms; however, 10% can suffer serious complications, such as acute respiratory failure (ARF) and admission to the intensive care unit (ICU). Predictors for the progression of SC have not yet been established, and conventional scoring systems for ICU patients are insufficient to predict severity. We aimed to identify simple and robust indicators to predict aggressive behaviors of SC. We evaluated 91 patients with SC and 81 non-SC patients who were admitted to the ICU, and 32 cases from the public functional genomics data repository for gene expression analysis. We analyzed the relationships between several predictors and clinicopathological characteristics in patients with SC. We performed gene set enrichment analysis (GSEA) to identify SC-specific gene sets. The acid-base imbalance (ABI), measured 24 h before serious complications, was higher in patients with SC than in non-SC patients. A high ABI was associated with an increased incidence of ARF, leading to mechanical ventilation and worse survival. GSEA revealed that SC correlated to gene sets reflecting inflammation/apoptotic response and airway inflammation. ABI can be used to indicate ARF in patients with SC and assist with early detection.

## 1. Introduction

Scrub typhus, caused by *Orientia tsutsugamushi*, is a zoonotic infectious disease transmitted by the bite of larvae of several species of *Leptotrombidium trombiculid* mites [1]. The acute febrile illness caused by vasculitis is a serious public health problem in south-east Asia, Australia, and islands in the Western Pacific and Indian Oceans. It threatens one billion people globally, and induces illness in one million people annually [2]. The clinical manifestations vary in severity, from a mild and self-limiting flu-like syndrome to a life-threatening disease [3,4].

The diverse pathologic changes in multiple organs are mainly due to focal or disseminated multi-organ vasculitis, or perivasculitis of small blood vessels, which show leukocyte-rich infiltration [4]. Patients who do not receive appropriate treatment often have severe and potentially fatal complications, such as sepsis, pneumonia, acute respiratory failure (ARF), acute kidney injury, shock, gastrointestinal bleeding, myocarditis, encephalitis, and disseminated intravascular coagulation, that can often be fatal [5,6,7,8,9].

Critically ill patients with severe infection may require intensive care due to severe disease complications. The mortality rate of the intensive care unit (ICU) due to scrub typhus varies from 3.5% to 30.3%, depending on subjects of previous studies [10,11,12,13]. Among patients requiring intensive care, the major complication is ARF leading to the requirement for mechanical ventilation (MV). In intensive care units (ICUs), the mortality of patients with ARF may be linked to the timing of MV application [14]. Therefore, identification of the predictors of ARF, as a severe complication in patients with scrub typhus, is crucial for the development of therapeutic strategies to increase survival. For patients who receive critical care, various conventional scoring systems have been established as indicators of mortality and/or complication rates, including the National Early Warning Score (NEWS), Acute Physiology and Chronic Health Evaluation (APACHE) II, and Sepsis Organ Failure Assessment (SOFA) [15,16,17]. These conventional scoring systems tend to be complex and subjective in clinical applications, making it difficult to predict clinical behavior and prepare therapeutic plans. Thus, there is a need for a simplified and robust indicator, to improve the prognostic and therapeutic performance in patients with scrub typhus.

Bioinformatic computational methods have recently been published to identify disease-specific molecular profiles within gene expression profiles [18,19]. Multiple computational tools have been developed to help identify potential indications for specific treatment using gene expression profiles that are available in gene expression omnibus (GEO) databases, which archive the results of a variety of rapidly-evolving, large-scale functional genomic experiments [20]. Gene set enrichment analysis (GSEA) allows for the efficient extraction of biological insights from long lists of differentially expressed genes by interrogating them at a systems level; this can help in the identification of key indicators or predictors of fatal complications [21]. However, studies of scrub typhus have not yet assessed the clinical application of GSEA from GEO.

The aim of this study was to identify simple indicators that could predict severe complications and mortality in patients with scrub typhus who are admitted to the ICU, and consequently improve the survival rate. In addition, we investigated specific gene sets associated with scrub typhus using the GSEA of GEO.

## 2. Materials and Methods

### 2.1. Patient Selection and Clinical Laboratory Parameters

This retrospective study included 91 patients with scrub typhus and 81 non-scrub typhus patients who were admitted to the ICU of Eulji University Hospital. The patients with scrub typhus were diagnosed based on clinical manifestations and serological tests results (indirect immunofluorescent antibody titer at more than four-fold) between May 2004 and February 2016. The non-scrub typhus patients were defined as patients admitted to the ICU for respiratory care from the emergency room between March 2015 and February 2016. Patients with MV prior to the admission to the ICU, do-not-resuscitate status, malignancy, and those who transferred to other hospitals were excluded. Supplementary Table S1 shows the criteria for admitting to the ICU in this study setting. Blood samples were analyzed using a standard based arterial blood gas analyzer (GEM^®^ Premier™ 3500, Werfen IL, Boston, MA, USA) that underwent daily calibration and quality control checks.

The clinical laboratory data collected from medical records included patient age, sex, comorbidity, reason for ICU admission, rash, eschar, systolic blood pressure, respiratory rate (RR), urine output, C-reactive protein level, Glasgow Coma Scale (GCS) score, and ICU length of stay. Acid-base imbalance (ABI), according to base excess or deficit in arterial blood gas analysis, was defined as the level of ABI. ARF was defined as a condition requiring clinical interventions (MV, nasal prong, facial mask) due to oxygen deficiency. The laboratory findings were measured 24 h before MV was performed.We evaluated conventional scoring systems such as the NEWS, APACHE II, and SOFA. The NEWS was evaluated by aggregating six physiological measurements: RR, oxygen saturation, temperature, systolic blood pressure, pulse rate, and level of consciousness [22]. The APACHE II score was calculated from 12 routine physiologic measurements: Age, alveolar-arterial oxygen gradient (AaDO_2_) or partial pressure of oxygen (PaO_2_), temperature, mean arterial pressure, arterial pH, heart rate, RR, serum sodium, serum potassium, creatinine, hematocrit, white blood cell count, and GCS. In addition, the APACHE II score used information relating to previous health status, including recent surgery, history of severe organ insufficiency, and immunocompromised state [23]. The SOFA score was calculated from six different scores: PaO_2_/fraction of inspired oxygen (FiO_2_), mean arterial pressure or administration of vasopressors, bilirubin, platelet count, creatinine or urine output, and GCS [24].

This study was approved by the Institutional Review Board of Eulji University Hospital (IRB No. 2016-02-006-001). Informed consent was waived by the board due to the retrospective nature of the study. This study was performed in accordance with the ethical standards of the Declaration of Helsinki, as revised in 2008.

### 2.2. Data Extraction and GSEA from the GEO Database

Two GEO datasets of gene expression in scrub typhus were downloaded from the domain training data (http://www.ncbi.nlm.nih.gov/geo/): GSE 24247 (Agilent-014850 Whole Human Genome Microarray) and GSE 16463 (Illumina human-6 v2.0 expression beadchip) [25]. The datasets included mRNA expression levels in monocytes infected by *Orientia tsutsugamushi* or other microorganisms extracted from infected patients.

GSEA is a method of analyzing and interpreting microarray and other data based on biological information. These biological sets contain published information about a biochemical pathway or coexpression obtained in a previous experiment. GSEA was performed using GSEA version 3.0 from the Broad Institute at MIT and Harvard (http://www.broadinstitute.org/gsea/index.jsp) [21].

The resources used for the analysis were as follows. The dataset included 17,810 features from the Molecular Signatures Database (MSigDB) v6.2. A gene set database (c2.cp.kegg.v6.2symbols.gmt), including 186 gene sets, was used for GSEA to identify host-damaging factors that were significantly enriched in genes associated with scrub typhus; for this analysis, 1000 permutations were used to calculate the *p*-values, and the permutation type was set to phenotype. We defined meaningful gene sets as those with a false discovery rate (FDR) of <0.25, and *p* < 0.05.

### 2.3. Statistical Analysis

Correlations between clinical laboratory parameters were analyzed using the Chi-square test, Student’s *t*-test, and Pearson correlation. Survival curves were generated by the Kaplan–Meier method, and the results were compared using log-rank tests. *p*-values less than 0.05 were considered statistically significant. Statistical analysis was conducted using R packages (http://www.r-project.org/) and IBM SPSS Statistics for Windows, version 24.0 (IBM, Corp., Armonk, NY, USA).

## 3. Results

### 3.1. Clinical Laboratory Findings of Scrub Typhus

**Figure 1:**
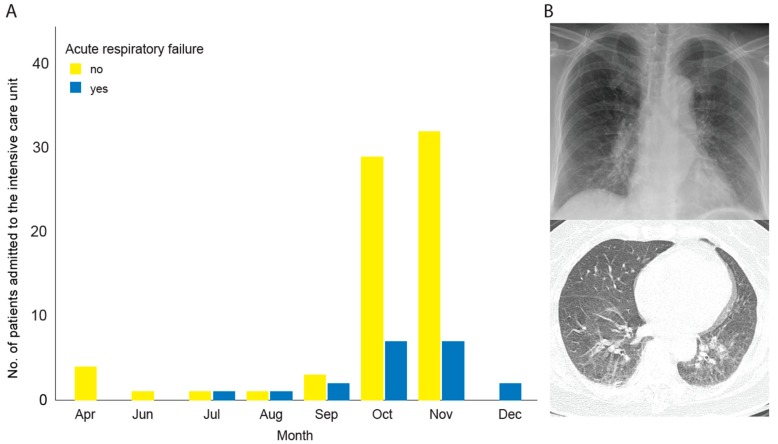
(**A**) Scrub typhus: Seasonal trends according to the month of admission (yellow: No association with acute respiratory failure; blue: Association with acute respiratory failure); (**B**) chest radiography in a 44-year old woman shows redistribution of pulmonary vasculature, peribronchial cuffing, and reticular and ground-glass opacity (top). Axial CT image obtained with lung window settings shows ground-glass opacity, centrilobular nodules, interlobular septal thickening, and scanty pleural effusion (bottom).

The number of patients with scrub typhus admitted to the ICU varied according to the season, with 36 cases in October (39.6%), and 39 in November (42.9%); the incidence of ARF was 36 cases (39.6%) (Figure 1). The median age of these 91 patients was 73 years (range, 31–94 years), and the male-to-female ratio was 44:47. The comorbidities included hypertension (44 cases, 48.4%), diabetes mellitus (25 cases, 27.5%), cerebrovascular disease (12 cases, 13.2%), and chronic liver disease (7 cases, 7.7%); 29 cases (31.9%) had no comorbidities. The major reason for ICU admission was sepsis (29 cases, 31.9%), followed by neurologic problems (24 cases, 26.4%), gastrointestinal bleeding (11 cases, 12.1%), cardiac problems (8 cases, 8.8%), and acute kidney injury (2 cases, 2.2%) (Table 1). The average scores for the conventional systems were as follows: NEWS, 9.8 ± 3.7; APACHE II, 15.4 ± 6.1; SOFA, 6.1 ± 3.1; and GCS, 12.6 ± 3.0.

In comparisons between the scrub typhus and non-scrub typhus groups, the scrub typhus group revealed prolonged ICU stay, as well as higher scores for conventional systems (NEWS, APACHE II, SOFA, GCS), compared to non-scrub typhus groups (all *p* < 0.05). Notably, ABI was higher in the scrub typhus group than the other (*p* = 0.012) (Table 1).

**Figure 2:**
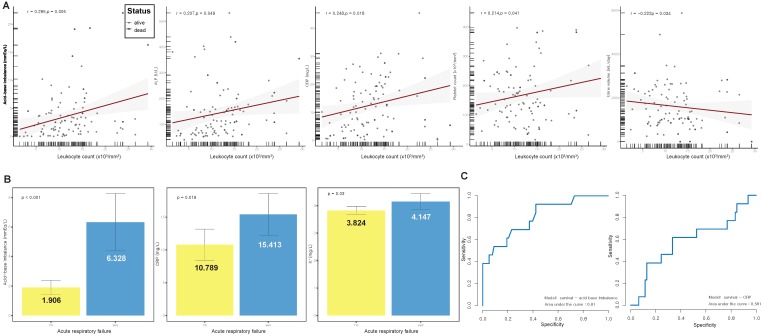
(**A**) Scatter plot of acid base-imbalance, alkaline phosphatase (ALP), C-reactive protein (CRP), platelet count, and 24-h urine output against leukocyte count; (**B**) comparisons of patients with and without acute respiratory failure show increased acid-base imbalance, CRP, and K^+^ in patients with acute respiratory failure; (**C**) receiver operating characteristic (ROC) curve showing the statistical performance of acid-base imbalance and CRP according to survival rate.

In the scrub typhus group, we evaluated the association between laboratory parameters and leukocyte count, which is a useful indicator of the severity of disease. The evaluated parameters included ABI, aminotransferase (ALT), aspartate aminotransferase (AST), alkaline phosphatase (ALP), lactate dehydrogenase (LDH), bilirubin, C-reactive protein (CRP), blood urea nitrogen (BUN), creatinine, PaO_2_, 24-h urine output, platelet, Na, K, and hematocrit. A high leukocyte count was related to increased ABI, platelet count, ALP, and CRP, and decreased urine output (*r* = 0.295, 0.214, 0.207, 0.248, and −0.222, respectively; all *p* < 0.05) (Figure 2A). In the comparison between laboratory parameters and ARF, which is a serious complication, ABI, CRP, and K were higher in patients with ARF than in those without ARF (all *p* < 0.05) (Figure 2B). ABI and CRP were simultaneously associated with an elevated leukocyte count and ARF in patients with scrub typhus. We used receiver operating characteristic (ROC) curves to evaluate the performance of survival models for ABI and CRP. The prognostic significance of ABI (92.3% sensitivity, 0.810 area under ROC) was superior to that of CRP (61.5% sensitivity, 0.587 area under ROC) (Figure 2C). In addition, the prognostic significance of ABI was superior to that of NEWS (54.3% sensitivity, 0.543 area under ROC), APACHE II (76.9% sensitivity, 0.771 area under ROC), SOFA (61.5% sensitivity, 0.668 area under ROC), GCS (60.3% sensitivity, 0.684 area under ROC), and leukocyte count (76.9% sensitivity, 0.661 area under ROC) (Supplementary Figure S1).

### 3.2. Clinical Manifestations of ABI

**Figure 3:**
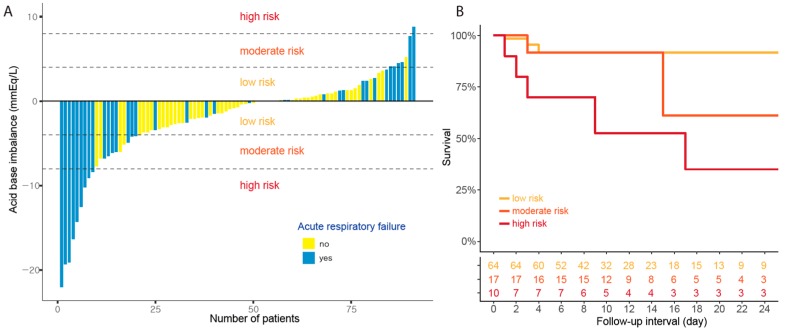
(**A**) Waterfall plot showing the acid-base imbalances of individual patients as the magnitude of change at admission to the ICU for scrub typhus; the amplitude of the bars in the waterfall plot indicate the degree of acid-base imbalance (yellow: Absence of acute respiratory failure; gray: Presence of acute respiratory failure); (**B**) survival analysis according to the risk groups based on acid-base imbalance; there are significant survival differences between the low-risk and moderate/high risk groups (*p* = 0.038).

The ABI cut-off was determined using a decision tree; the target attribute was set as ARF application. Based on the optimal cut-off using the above method, ABI was classified into three risk groups as follows: Low (−4 ≥ ABI ≤ 4; 64 cases, 70.3%), moderate (4 < ABI ≤ 8 or −4 > ABI ≥ −8; 17 cases, 18.7%), and high (ABI > 8 or ABI < −8; 10 cases, 11.0%) (Supplementary Figure S2). Of the 64 low-risk patients, 14 (21.9%) had ARF. Of the 17 patients with moderate risk, 12 (70.6%) had ARF. All 10 high-risk patients (100%) had ARF (Figure 3A).

We analyzed the differences in clinical laboratory parameters according to high ABI. Comparisons between the low-risk and moderate/high-risk ABI groups showed that moderate/high risk was associated with an increased ICU stay, MV, and blood transfusion (all *p* < 0.05). Gastrointestinal bleeding was frequently observed in the moderate/high-risk group, compared to that in the low-risk group (*p* = 0.023). The scores for conventional systems (NEWS, APACHE II, SOFA, GCS) were higher in the moderate/high-risk group than those in the low-risk group (all *p* < 0.05). The moderate/high-risk group was also associated with poor survival compared to that in the low-risk group (*p* = 0.038) (Figure 3B) (Table 2). After controlling for confounders, including NEWS, APACHE II, SOFA, and GCS scores, there was still a significant survival difference between the low-risk and moderate/high-risk groups (HR, 5.2; 95% CI, 1.16–23.29; *p* = 0.037).

### 3.3. Significant Gene Sets Associated with Scrub Typhus

**Figure 4:**
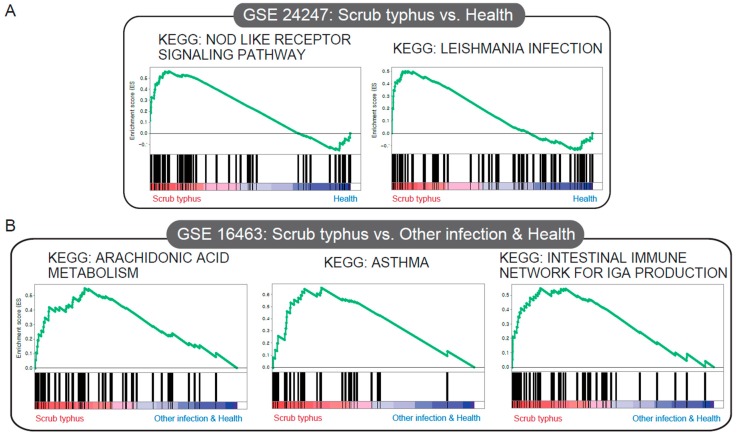
Gene expression data analyzed using gene set enrichment analysis (GSEA) to extract biological information. In each thumbnail, the green curve represents the evolution of the density of the genes identified in the RNA-Seq data. GSEA calculates these by walking down the ranked-ordered list of genes, increasing a running-sum statistic when a gene is in the gene set, and decreasing it when it is not. (**A**) KEGG JAK–STAT signaling pathway, KEGG NOD-like receptor signaling pathway, compared to the healthy group (GSE24247). (**B**) KEGG arachidonic acid metabolism, KEGG asthma, and KEGG intestinal immune network for IGA production, compared to other infection (GSE16463).

We conducted GSEA to identify gene sets associated with scrub typhus in GSE 24247 (four cases with scrub typhus, and four control cases) and GSE 16463 (four cases with scrub typhus, seven cases with dengue fever, seven cases with murine typhus, four cases with malaria, and two control cases). We identified two significantly enriched gene sets linked to inflammation/apoptotic response (KEGG NOD-like receptor signaling pathway) and protective immunity against host-adapted organisms (KEGG Leishmania infection) in the scrub typhus group of GSE 24247 (Figure 4A) (Supplementary Table S2). Comparison of scrub typhus and non-scrub typhus of GSE 16463 revealed that scrub typhus was associated with airway inflammation (KEGG asthma), cytochrome P450 pathways (KEGG arachidonic acid metabolism), and a first-line defense mediated by immunoglobulin A (IgA) antibodies (KEGG intestinal immune network for IGA production) (Figure 4B) (Supplementary Table S3).

## 4. Discussion and Conclusions

The pathogenesis of *Rickettsia* spp. is associated with the production of cytokines by infected endothelial cells and endothelial proliferation, which lead to a vasculitis-like syndrome. Scrub typhus appears, at least early in the infection, to target dendritic cells, neutrophils, leukocytes, and macrophages rather than endothelial cells, with cell invasion mediated by fibronectin [26]. Scrub typhus is usually an uncomplicated febrile illness with fever, skin rash, headache, myalgia, and conjunctivitis, and is easily treated with doxycycline and/or tetracycline [27]. However, serious infection-related vital organ failure, including the lungs, brain, heart, and kidneys, as well as disseminated coagulopathy, is often fatal [4,9]. In our study, the relationship between high ABI and scrub typhus can be considered as a unique indicator in the progression of scrub typhus associated ARF. In addition, the use of MV to overcome ARF due to ABI will most likely lead to a prolonged ICU stay, and higher scores of conventional surveillance systems. Using the change of ABI appearing before proceeding to ARF, active treatment such as MV can be performed in order to improve survival in patients with scrub typhus with aggressive behavior.

In the host response to microorganisms, clinical manifestations may result from the systemic release of cytokines, as well as inflammatory mediators [28]. Indeed, de Fost, et al. suggested that cell-mediated immunity was vital in controlling scrub typhus infection [29]. However, high levels of immune-related cytokines could lead to severe illness, organ damage, and death [30]. In published data, ABI was significantly associated with changes in the inflammatory response [31,32,33]. Severe organ damage can induce physiological changes by alteration of the host metabolic pathways and activation of the immune system [34,35]. In other words, the infection-associated injury could induce tissue acidosis, which is reflected in ABI as base excess or base deficit [36]. Other studies have reported scrub typhus-induced vasculitis to be related to ABI [37,38,39,40,41,42].

In patients with scrub typhus, we identified five gene sets from GEO data; these included asthma and the immune response. The results suggested that scrub typhus infection is related to more aggressive respiratory dysfunction and inflammatory response, when compared to healthy patients or those with other infection. Our results showed that ABI was related to leukocyte count and ARF, which reflect immune/inflammation response and respiratory dysfunction, respectively. Higher ABI was also significantly correlated with unfavorable clinicopathological parameters, and poor survival compared to those for lower ABI. Furthermore, ABI was increased in patients with high conventional scores such as NEWS, SOFA, and GCS for predicting worse clinical outcome. There was a significant relationship between ABI and APACHE II, but it is difficult to give clinical significance since the APACHE II score included the arterial pH. 

In clinical practice, Arterial Blood Gas Analysis (ABGA) is a highly sensitive, rapid, and simple method for the diagnosis of ABI according to base excess or deficit. Abdul–Malak, et al. showed that ABI could alter the inflammatory response in a number of ways, including scavenging and modulation of cytokine production [43]. Another study reported ABI to be a useful predictor of septic shock as well as severe sepsis [44]. ABI was also associated with severe complications such as mortality [45,46,47], intra-abdominal injury [48,49], and transfusion [50], as well as failed weaning attempts [51]. The severe complications of scrub typhus include jaundice, meningoencephalitis, myocarditis, interstitial pneumonia leading on to ARF, and renal failure [3,52]. Although MV for ARF is a well-recognized reason for ICU support, it can be difficult to predict the need for MV support. Our results showed an increased rate of MV application and mortality in patients with a higher ABI. The ABI may be an important point for predicting MV application because the early diagnosis and management of ARF combined with appropriate MV support aids prognosis.

There are some limitations that should be acknowledged in this study. The relationship between mortality (including serious complications) and ABI could not be conclusively proven due to the cross-sectional design of this study. Several clinical conditions, such as respiratory acidosis, hypercapnia, and hypoxia could induce ABI. With regards to the detection of risk factors, prospective studies should be conducted to identify the continuous associations among these factors over time. The people enrolled in this study had serious clinical implications, such as elevated leukocyte counts, multiple organ complications, and underlying diseases that can affect mortality. In the present study, these risk factors associated with ABI and ARF (including indication of MV) were overlapping; hence, it was difficult to make accurate conclusions. To complement these points, we performed GSEA to clarify the specific pathological manifestations of scrub typhus: Immune response and respiratory disease. However, the relationship between these variables remained unclear.

In summary, the study showed that higher ABI was associated with unfavorable clinical parameters and poor prognosis, helping to predict MV application among patients with scrub typhus admitted to the ICU. The ABI can be used as an indicator for an early diagnosis of the severity of disease in patients with scrub typhus. In the future, prospective large-scale studies using molecular serotyping are required to confirm the relationship between ABI and poor clinical outcomes.

## Figures and Tables

**Table 1:** Comparison between the 91 scrub typhus patients and the 81 non-scrub typhus patients admitted to the intensive care unit.

Parameters	Scrub Typhus (*n* = 91)	Non-Scrub Typhus (*n* = 81)	*p*-Value (*χ*^2^)
**Clinical manifestation**			
Age, years	70.9 ± 10.9	69.7 ± 13.3	0.514 ^1^
Sex, male, %	44 (48.4)	55 (67.9)	**0.010**
**Critical care**			
ICU length of stay (day)	6.1 ± 8.5	3.7 ± 2.0	**0.013 ^1^**
Mechanical ventilation, %	26 (28.6)	2 (2.5)	**<0.001 ^2^**
Blood transfusion, %	22 (24.2)	7 (8.6)	**0.007**
**Reasons for ICU admission**			
Sepsis, %	29 (31.9)	29 (35.8)	0.586
Neurologic problem, %	24 (26.4)	5 (6.2)	**<0.001**
Gastrointestinal bleeding, %	11 (12.1)	0	**0.001 ^2^**
Cardiac problem, %	8 (8.8)	2 (2.5)	0.077 ^2^
Acute kidney injury, %	2 (2.2)	0	0.180 ^2^
Pulmonary thromboembolism, %	0	23 (28.4)	**<0.001 ^2^**
Hemoptysis, %	0	15 (18.5)	**<0.001 ^2^**
**Comorbidity**			
Hypertension, %	44 (48.4)	40 (49.4)	0.893
Diabetes mellitus, %	25 (27.5)	19 (23.5)	0.547
Cerebrovascular disease, %	12 (13.2)	14 (17.3)	0.454
Chronic liver disease, %	7 (7.7)	4 (4.9)	0.461 ^2^
**Conventional scoring systems**			
NEWS	9.8 ± 3.7	5.6 ± 2.7	**<0.001 ^1^**
APACHE II	15.4 ± 6.1	12.0 ± 4.9	**<0.001 ^1^**
SOFA	6.1 ± 3.1	3.2 ± 2.0	**<0.001 ^1^**
GCS	12.6 ± 3.0	14.1 ± 1.8	**<0.001 ^1^**
**Acid Base Imbalance**	3.7 ± 4.4	2.4 ± 1.7	**0.012 ^1^**

ICU, intensive care unit; NEWS, National Early Warning Score; APACHE, Acute Physiology and Chronic Health Evaluation; SOFA, Sepsis Organ Failure Assessment; GCS, Glasgow Coma Scale; ^1^ Student’s *t*-test; ^2^ Fisher’s exact test; *p* < 0.05 is shown in bold.

**Table 2:** Comparisons between acid-base imbalance and clinical laboratory parameters in 91 patients admitted to the intensive care unit with scrub typhus.

Parameters	Low Risk (*n* = 64)	Moderate Risk (*n* = 17)	High Risk (*n* = 10)	*p*-Value ^1^ (*χ*^2^)
**Clinical manifestation**				
Age, years	70.4 ± 11.8	74.4 ± 6.7	68.2 ± 10.4	0.5 ^2^
Sex, male, %	31 (48.4)	6 (35.3)	7 (70.0)	0.999
Rash, %	16 (25.0)	1 (5.9)	1 (10.0)	0.102
Eschar, %	31 (48.4)	7 (41.2)	3 (30.0)	0.443
**Critical care**				
ICU length of stay (day)	4.3 ± 4.9	7.7 ± 6.9	14.7 ± 18.9	**0.026 ^2^**
Mechanical ventilation, %	5 (7.8)	11 (64.7)	10 (100)	**<0.001**
Blood transfusion, %	11 (17.2)	6 (35.3)	5 (50.0)	**0.033**
**Reasons for ICU admission**				
Sepsis, %	23 (35.9)	3 (17.6)	3 (30.0)	0.3
Neurologic problem, %	21 (32.8)	2 (11.8)	1 (10.0)	0.059
Gastrointestinal bleeding, %	4 (6.3)	4 (23.5)	3 (30.0)	**0.023**
Cardiac problem, %	5 (7.8)	2 (11.8)	1 (10.0)	0.918
Acute kidney injury, %	1 (1.6)	1 (5.9)	0	0.999
**Comorbidity**				
Hypertension, %	29 (45.3)	9 (52.9)	6 (60.0)	0.507
Diabetes mellitus, %	18 (28.1)	3 (17.6)	4 (40.0)	0.999
Cerebrovascular disease, %	8 (12.5)	1 (5.9)	3 (30.0)	0.999
Chronic liver disease, %	6 (9.4)	0	1 (10.0)	0.619
**Conventional scoring systems**				
NEWS	9.0 ± 3.4	12.0 ± 4.2	11.4 ± 3.4	**0.001 ^2^**
APACHE II	14.4 ± 5.2	16.1 ± 6.3	20.6 ± 8.4	**0.038 ^2^**
SOFA	5.5 ± 2.6	6.4 ± 3.4	9.3 ± 3.5	**<0.02 ^2^**
GCS	13.1 ± 2.4	12.1 ± 3.0	10.2 ± 5.2	**0.044 ^2^**
**ICU mortality, %**	4 (6.3)	3 (17.6)	6 (60.0)	**0.001**

ICU, intensive care unit; NEWS, National Early Warning Score; APACHE, Acute Physiology and Chronic Health Evaluation; SOFA, Sepsis Organ Failure Assessment; GCS, Glasgow Coma Scale; ^1^ low risk versus moderate/high risk; ^2^ Student’s *t*-test; *p* < 0.05 is shown in bold.

## Supplementary Materials

The following are available online at https://www.mdpi.com/2077-0383/8/10/1580/s1, Figure S1: A Receiver operating characteristic (ROC) curve showing the statistical performance of (**A**) NEWS, (**B**) APACHE II, (**C**) SOFA, (**D**) GCS and (**E**) leukocyte count according to survival rate; Figure S2: A decision tree of acid-base imbalance to predict acute respiratory failure in 91 patients with scrub typhus (yellow: Absence of acute respiratory failure; blue: Presence of acute respiratory failure); Table S1: The criteria for admission to intensive care unit; Table S2: Gene sets within the top 10-ranked list related to scrub typhus (scrub typhus versus health, GSE 24247); Table S3: Gene sets within the top 10-ranked list related to scrub typhus (scrub typhus versus other infection, GSE 16463).
