# Supplementary material for: Higher Acid-Base Imbalance Associated with Respiratory Failure Could Decrease the Survival of Patients with Scrub Typhus during Intensive Care Unit Stay: A Gene Set Enrichment Analysis
Source: J Clin Med. 2019 Oct 2;8(10):1580. doi: 10.3390/jcm8101580 (PMC6832163; doi:10.3390/jcm8101580)
Supplement: Supplementary file 1 [file jcm-08-01580-s001.pdf]

Supplementary information

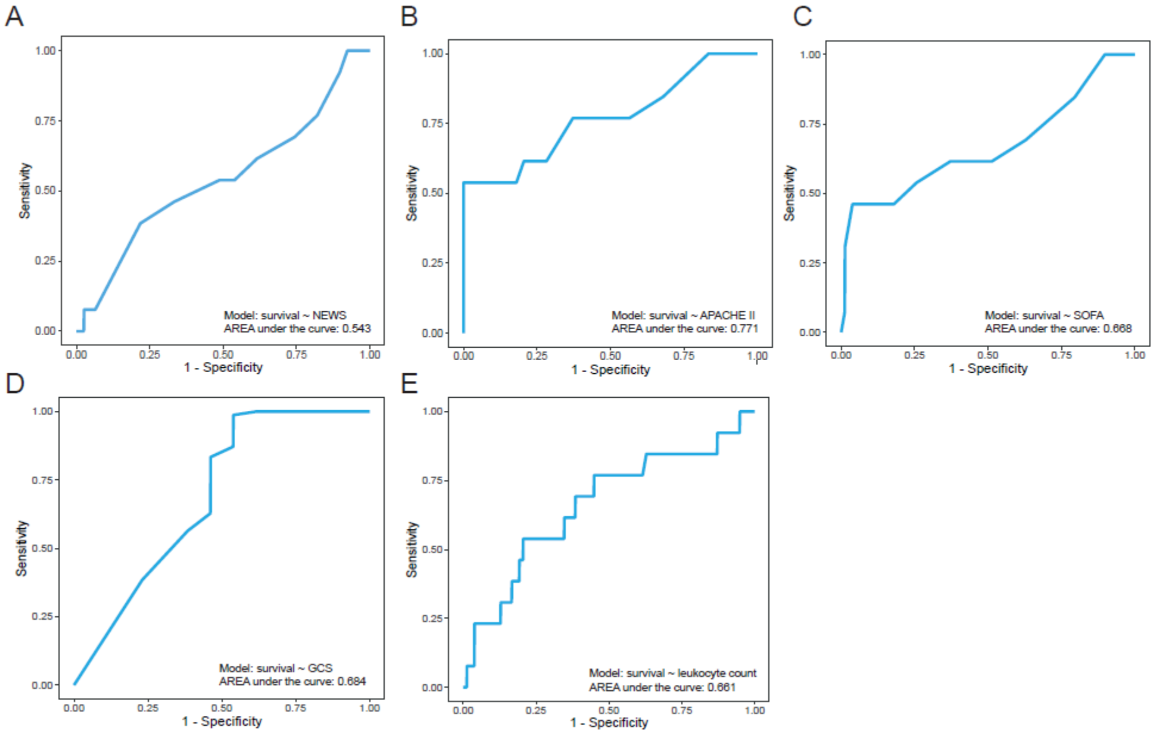

Supplementary figure 1

Supplementary figure 1. A Receiver operating characteristic (ROC) curve showing the statistical performance of (A) NEWS, (B) APACHE II, (C) SOFA, (D) GCS and (E) leukocyte count according to survival rate.

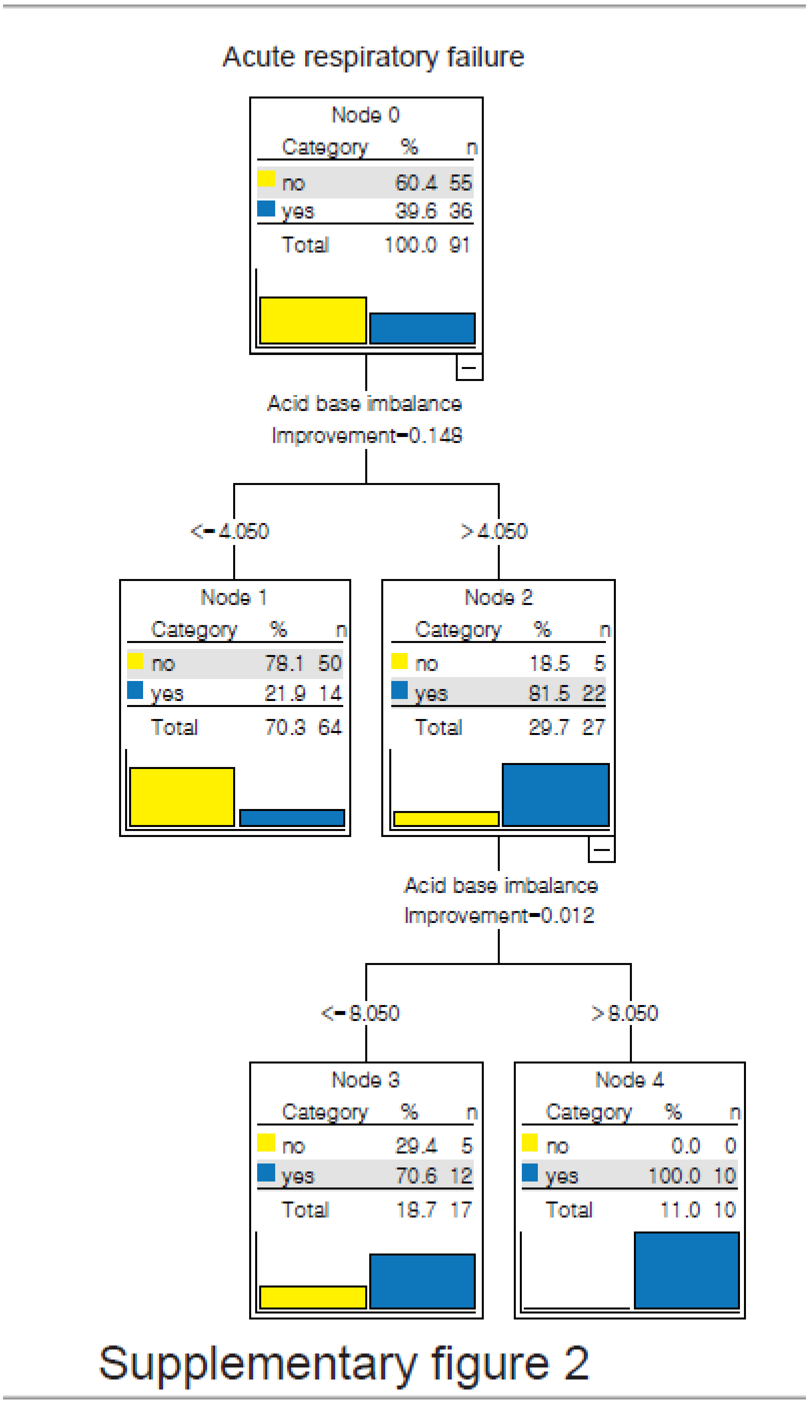

Supplementary figure 2. A decision tree of acid-base imbalance to predict acute respiratory failure in 91 patients with scrub typhus (yellow: absence of acute respiratory failure; blue: presence of acute respiratory failure)

Supplementary table 1. The criteria for admission to intensive care unit

**Vital signs**
Pulse rate<40 times/minute or >150 times/minute
Systolic blood pressure<80 mmHg
Diastolic blood pressure>120 mmHg
Average arterial pressure<60 mmHg
More than 35 breaths/min
**Laboratory tests**
Serum sodium<110 mEq/L or >170 mEq/L
Serum potassium below 2.0 mEq/L or above 7.0 mEq/L
Arterial blood oxygen partial pressure<50 mmHg
Arterial pH<7.1 or >7.7
Blood glucose level>800 mg/dl
Serum calcium>15 mg/dl
Toxic doses of drugs or chemicals that may be of hemodynamic or neurological problem
**Radiology/Ultrasonography/Computed Tomography**
Cerebral bleeding, or subarachnoid hemorrhage with changes in consciousness or local neurological signs
Hemodynamically unstable rupture of the intestines, bladder, liver, esophagus, varicose veins or uterus
Aortic dissection
**Echocardiogram**
Myocardial infarction associated with complex arrhythmia, hemodynamic instability or congestive heart failure
Continuous ventricular tachycardia or ventricular fibrillation
Complete atrioventricular block with hemodynamic instability
**Acute onset physical signs**
When the pupil size is different in an unconscious patient
Anuria
Airway obstruction
Coma
Persistent convulsions
Cardiac tamponade

Supplementary table 2. Gene sets within the top 10-ranked list related to scrub typhus (scrub typhus versus health, GSE 24247)

NAME	SIZE	ES	NES	NOM (p-value)	FDR (q-value)	FWER (p-value)
KEGG_RIG_I_LIKE_RECEPTOR_SIGNALING_PATHWAY	66	**0.702**	**1.593**	**< 0.001**	**0.396**	0.29
KEGG_TOLL_LIKE_RECEPTOR_SIGNALING_PATHWAY	96	0.627	1.541	< 0.001	0.358	0.483
KEGG_APOPTOSIS	87	0.565	1.522	< 0.001	0.333	0.64
KEGG_JAK_STAT_SIGNALING_PATHWAY	150	0.592	1.516	< 0.001	0.269	0.679
**KEGG_NOD_LIKE_RECEPTOR_SIGNALING_PATHWAY**	**62**	**0.562**	**1.513**	**< 0.001**	**0.221**	**0.689**
**KEGG_LEISHMANIA_INFECTION**	**70**	**0.501**	**1.504**	**< 0.001**	**0.205**	**0.733**
KEGG_CYTOSOLIC_DNA_SENSING_PATHWAY	49	0.744	1.436	0.071	0.386	0.86
KEGG_PRION_DISEASES	35	0.546	1.432	< 0.001	0.358	0.877
KEGG_CYTOKINE_CYTOKINE_RECEPTOR_INTERACTION	257	0.599	1.427	< 0.001	0.337	0.877
KEGG_INTESTINAL_IMMUNE_NETWORK_FOR_IGA_PRODUCTION	48	0.534	1.427	< 0.001	0.304	0.877
KEGG_RIG_I_LIKE_RECEPTOR_SIGNALING_PATHWAY	66	0.702	1.593	< 0.001	0.396	0.29

ES: Enrichment score, NES: Normalized enrichment score, FDR: False discovery rate, NOM: Nominal p-value, FDR: False discovery rate, FWER: Family-wise error rate

Supplementary table 3. Gene sets within the top 10-ranked list related to scrub typhus (scrub typhus versus other infection, GSE 16463)

NAME	SIZE	ES	NES	NOM (p-value)	FDR (q-value)	FWER (p-value)
**KEGG_ARACHIDONIC_ACID_METABOLISM**	**53**	**0.551**	**1.834**	**0.002**	**0.143**	**0.105**
**KEGG_ASTHMA**	**30**	**0.656**	**1.784**	**0.010**	**0.139**	**0.172**
**KEGG_INTESTINAL_IMMUNE_NETWORK_FOR_IGA_PRODUCTION**	**48**	**0.549**	**1.710**	**0.009**	**0.222**	**0.302**
KEGG_ETHER_LIPID_METABOLISM	28	0.486	1.633	0.006	0.367	0.494
KEGG_GLUTATHIONE_METABOLISM	46	0.501	1.537	0.050	0.653	0.721
KEGG_ALPHA_LINOLENIC_ACID_METABOLISM	17	0.489	1.506	0.022	0.679	0.772
KEGG_DRUG_METABOLISM_CYTOCHROME_P450	71	0.404	1.504	0.046	0.591	0.779
KEGG_TYPE_I_DIABETES_MELLITUS	44	0.462	1.501	0.051	0.530	0.786
KEGG_EPITHELIAL_CELL_SIGNALING_IN_HELICOBACTER_PYLORI_INFECTION	65	0.408	1.493	0.042	0.499	0.799
KEGG_CYTOKINE_CYTOKINE_RECEPTOR_INTERACTION	254	0.397	1.473	0.035	0.510	0.83
KEGG_ARACHIDONIC_ACID_METABOLISM	53	0.551	1.834	0.002	0.143	0.105

ES: Enrichment score, NES: Normalized enrichment score, FDR: False discovery rate, NOM: Nominal p-value, FDR: False discovery rate, FWER: Family-wise error rate
